# *p*,*p*′-DDE activates CatSper and compromises human sperm function at environmentally relevant concentrations

**DOI:** 10.1093/humrep/det372

**Published:** 2013-09-24

**Authors:** Renata S. Tavares, Steven Mansell, Christopher L.R. Barratt, Stuart M. Wilson, Stephen J. Publicover, João Ramalho-Santos

**Affiliations:** 1 Biology of Reproduction and Stem Cell Group, CNC—Center for Neuroscience and Cell Biology, University of Coimbra, Coimbra 3001-401, Portugal; 2 Department of Life Sciences, University of Coimbra, Coimbra 3001-401, Portugal; 3 Medical Research Institute, Ninewells Hospital and Medical School, Dundee DD1 9SY, UK; 4 School of Biosciences, University of Birmingham, Birmingham B15 2TT, UK

**Keywords:** *p*,*p*′-DDE, intracellular Ca^2+^ concentration, Ca^2+^ influx, CatSper, acrosome reaction

## Abstract

**STUDY QUESTION:**

Is the environmental endocrine disruptor *p*,*p*′-dichlorodiphenyldichloroethylene (*p*,*p*′-DDE) able to induce non-genomic changes in human sperm and consequently affect functional sperm parameters?

**SUMMARY ANSWER:**

*p*,*p*′-DDE promoted Ca^2+^ flux into human sperm by activating CatSper channels even at doses found in human reproductive fluids, ultimately compromising sperm parameters important for fertilization.

**WHAT IS KNOWN ALREADY:**

*p*,*p*′-DDE may promote non-genomic actions and interact directly with pre-existing signaling pathways, as already observed in other cell types. However, although often found in both male and female reproductive fluids, its effects on human spermatozoa function are not known.

**STUDY DESIGN, SIZE, DURATION:**

Normozoospermic sperm samples from healthy individuals were included in this study. Samples were exposed to several *p*,*p*′-DDE concentrations for 3 days at 37°C and 5% CO_2_ *in vitro* to mimic the putative continuous exposure to this toxicant in the female reproductive tract *in vivo.* Shorter *p*,*p*′-DDE incubation periods were also performed in order to monitor sperm rapid Ca^2+^ responses. All experiments were repeated on a minimum of five sperm samples from different individuals.

**PARTICIPANTS/MATERIALS, SETTING, METHODS:**

All healthy individuals were recruited at the Biosciences School, University of Birmingham, the Medical Research Institute, University of Dundee and in the Human Reproduction Service at University Hospitals of Coimbra. Intracellular Ca^2+^ concentration ([Ca^2+^]_i_) was monitored by imaging single spermatozoa loaded with Oregon Green BAPTA-1AM and further whole-cell patch-clamp recordings were performed to validate our results. Sperm viability and acrosomal integrity were assessed using the LIVE/DEAD sperm vitality kit and the acrosomal content marker PSA-FITC, respectively.

**MAIN RESULTS AND THE ROLE OF CHANCE:**

*p*,*p*′-DDE rapidly increased [Ca^2+^]_i_ (*P* < 0.05) even at extremely low doses (1 pM and 1 nM), with magnitudes of response up to 200%, without affecting sperm viability, except after 3 days of continuous exposure to the highest concentration tested (*P* < 0.05). Furthermore, experiments performed in a low Ca^2+^ medium demonstrated that extracellular Ca^2+^ influx was responsible for this Ca^2+^ increase (*P* < 0.01). Mibefradil and NNC 55-0396, both inhibitors of the sperm-specific CatSper channel, reversed the *p*,*p*′-DDE-induced [Ca^2+^]_i_ rise, suggesting the participation of CatSper in this process (*P* < 0.05). In fact, whole-cell patch-clamp recordings confirmed CatSper as a target of *p*,*p*′-DDE action by monitoring an increase in CatSper currents of >100% (*P* < 0.01). Finally, acrosomal integrity was adversely affected after 2 days of exposure to *p*,*p*′-DDE concentrations, suggesting that [Ca^2+^]_i_ rise may cause premature acrosome reaction (*P* < 0.05).

**LIMITATIONS, REASONS FOR CAUTION:**

This is an *in vitro* study, and caution must be taken when extrapolating the results.

**WIDER IMPLICATIONS OF THE FINDINGS:**

A novel non-genomic *p*,*p*′-DDE mechanism specific to sperm is shown in this study. *p*,*p*′-DDE was able to induce [Ca^2+^]_i_ rise in human sperm through the opening of CatSper consequently compromising male fertility. The promiscuous nature of CatSper activation may predispose human sperm to the action of some persistent endocrine disruptors.

**STUDY FUNDING/COMPETING INTEREST(S):**

The study was supported by both the Portuguese National Science Foundation (FCT; PEst-C/SAU/LA0001/2011) and the UK Wellcome Trust (Grant #86470). SM was supported by the Infertility Research Trust. RST is a recipient of a PhD fellowship from FCT (SFRH/BD/46002/2008). None of the authors has any conflict of interest to declare.

## Introduction

Exposure to numerous environmental toxicants may have contributed to a decline in human semen quality, particularly in terms of sperm counts, reported worldwide (Carlsen *et al*., 1992). In particular, the so-called endocrine disruptors may influence male reproductive function by interfering with hormonal activity (Sharpe, 1995). *p*,*p*′-Dichlorodiphenyldichloroethylene (*p*,*p*′-DDE), a well-known environmental endocrine disruptor, is a persistent dichlorodiphenyl trichloroethane (DDT) metabolite often found in human reproductive fluids (Kumar *et al*., 2000; Dallinga *et al*., 2002; Younglai *et al*., 2002; Pant *et al*., 2004) that has been associated with failed fertilization (Younglai *et al*., 2002). Moreover, high levels of *p*,*p*′-DDE correlate with diminished standard semen parameters (Ayotte *et al*., 2001; De Jager *et al*., 2006; Toft *et al*., 2006; Aneck-Hahn *et al*., 2007), sperm viability (De Jager *et al*., 2006; Aneck-Hahn *et al*., 2007), enhanced sperm chromatin/DNA damage (De Jager *et al*., 2006) and altered accessory sex gland secretions (Ayotte *et al*., 2001; Pant *et al*., 2004). It should be noted, however, that current data includes contradictory results (Hauser *et al*., 2002, 2003; Rignell-Hydbom *et al*., 2005a, 2005b; Stronati *et al*., 2006). In rats, exposure to *p*,*p*′-DDE *in utero* and through lactation significantly decreased cauda epididymal sperm counts (Loeffler and Peterson, 1999) and affected anogenital distance and nipple retention, both accurate indicators of endocrine disruption (You *et al*., 1998; Loeffler and Peterson, 1999).

While most studies have focused on the long-term effects of *p*,*p*′-DDE, it has become clear that this compound may also promote rapid non-genomic actions, and interact directly with pre-existing signaling pathways. However, there is no data on such non-genomic effects in human spermatozoa. Calcium (Ca^2+^) is an intracellular messenger involved in several cellular events, and the amplitude, spatial and temporal features of Ca^2+^ signaling establish specific responses (Younglai *et al*., 2006). *p*,*p*′-DDE, related DDT metabolites and/or other pesticides have been shown to adversely affect function by interfering with Ca^2+^ signals in many cell types, including human placenta (Treinen and Kulkarni, 1986), granulosa-lutein cells (Younglai *et al*., 2004; Wu *et al*., 2006) and umbilical vein endothelial cells (HUVE; Younglai *et al*., 2006). Moreover, similar effects were reported not only in bovine oviductal (Tiemann *et al*., 1998) and rat myometrial and vascular smooth muscle cells (Juberg *et al*., 1995; Ruehlmann *et al*., 1998) but also in mice pancreatic β cells (Nadal *et al*., 2000) and in a pituitary tumor cell line (Wozniak *et al*., 2005).

Despite their small size and low cytoplasm content, sperm cells are equipped with extraordinary mechanisms capable of regulating intracellular Ca^2+^ concentration ([Ca^2+^]_i_) and production of complex Ca^2+^ signals (reviewed in Jimenez-Gonzalez *et al*. (2006)). In ejaculated spermatozoa, [Ca^2+^]_i_ was shown to control several key events (Eisenbach, 1999; Carlson *et al*., 2003; Spehr *et al*., 2003; Suarez and Ho, 2003; Alasmari *et al*., 2013) such as the acrosome reaction (AR; Kirkman-Brown *et al*., 2002), an exocytic process without which spermatozoa would be unable to successfully fertilize an oocyte (Ramalho-Santos *et al*., 2007). *In vitro* experiments conducted in a porcine model have shown that exposure to an organochlorine mixture containing *p*,*p*′-DDE increased cytosolic Ca^2+^ levels, possibly leading to an enhanced AR (Campagna *et al*., 2009).

The present work was carried out to determine whether *p*,*p*′-DDE at environmentally relevant concentrations modulates intracellular Ca^2+^ levels, and alters AR, thus potentially affecting human male fertility. Here, we report not only that *p*,*p*′-DDE raises [Ca^2+^]_i_ and stimulates AR but also that CatSper, a sperm-specific ion channel, is a target of *p*,*p*′-DDE.

## Materials and Methods

All reagents were provided by Sigma-Aldrich (St. Louis, MO, USA) unless stated otherwise. A 99.1% chemically pure *p*,*p*′-DDE was dissolved in dimethyl sulphoxide (DMSO) to a stock concentration of 62.88 mM.

### Human biological samples

Fresh normozoospermic sperm samples from both human healthy donors recruited at the Biosciences School, University of Birmingham, and Medical Research Institute, University of Dundee (Ethics number 08/S1402); as well as healthy patients undergoing routine semen analysis or fertility treatments in the Human Reproduction Service at University Hospitals of Coimbra were used accordingly to the proper ethical and Internal Review Board of the participating Institutions. All individuals signed informed consent forms. Samples were obtained by masturbation after 3–5 days of sexual abstinence and seminal analysis was performed according to the World Health Organization guidelines (WHO, 2010). All samples used in this study had no detectable leukocytes (or any other round cells) and presented >80% viable sperm after processing.

### Single-cell Ca^2+^ imaging experiments

After liquefaction, spermatozoa were prepared by direct swim-up and allowed to capacitate in a supplemented Earle's balanced salt solution (sEBSS) containing 1.8 mM CaCl_2_, 5.4 mM KCl, 0.81 mM MgSO_4_, 25.0 mM NaHCO_3_, 1.0 mM NaH_2_PO_4_, 116.4 mM NaCl, 5.5 mM d-glucose, 2.5 mM Na–pyruvate, 41.8 mM Na–lactate and 0.3% (w/v) BSA, for at least 3 h at 37°C under 5% CO_2_/95% air before starting imaging. [Ca^2+^]_i_ measurements were performed after loading 4 million/ml sperm with the Ca^2+^ fluorescent marker Oregon Green BAPTA-1AM (10 µM; Molecular Probes, Eugene, OR, USA) for 1 h at 37°C under 5% CO_2_/95% air, as described elsewhere (Mota *et al*., 2012). All experiments were carried out in a dark room at 25°C with a constant perfusion rate of 0.4 ml/min. Real-time recordings were performed at intervals of 2.5 s using an IQ acquisition software platform (Andor Technology, Belfast, UK). Analysis of images, background correction and normalization of data was performed as described previously (Kirkman-Brown *et al*., 2000). The region of interest was drawn around the posterior head and neck region of each cell and raw intensity values were imported into Microsoft Excel and normalized using the equation ▵*F* = [(*F* – *F*_basal_)/*F*_basal_] × 100%, where ▵*F* is % change in intensity at time *t*, *F* is fluorescence intensity at time *t* and *F*_basal_ is the mean basal *F* established in the beginning of each experiment before application of any stimulus. Each cell was considered to respond when the mean of 10 determinations of normalized *F* during the exposure period differed significantly from the mean of 10 determinations of normalized *F* during control (or inhibitor) treatment (*P* < 0.05). Mean amplitudes and percent responsive cells were calculated for each concentration in each sperm sample analyzed.

### Measurements of intracellular Ca^2+^ levels

To evaluate the effect of *p*,*p*′-DDE on [Ca^2+^]_i_, spermatozoa were exposed to a wide range of concentrations (1 pM–50 µM) diluted in standard sEBSS. To further assess the contribution of the internal Ca^2+^ stores, similar experiments were performed in a low-Ca^2+^ sEBSS medium (Ca^2+^ was adjusted to 5 and 6 mM EGTA was added; final [Ca^2+^] < 500 nM). Finally, inhibition studies were performed using 30 µM mibefradil and 10 µM NNC 55-0396 (Brenker *et al*., 2012). These drugs have been shown to effectively block CatSper currents at these concentrations. When a plateau in the *p*,*p*′-DDE-induced [Ca^2+^]_i_ rise was reached, either mibefradil or NNC 55-0396 was added, allowing the amplitudes of agonist and antagonist effects to be compared in each cell. Before finishing each experiment, spermatozoa were washed with standard sEBSS and exposed to 3.2 µM progesterone to determine if they were responding properly to the physiological stimuli (positive control). Solvent controls were carried out with 0.3% (v/v) DMSO.

### Whole-cell patch-clamp experiments

Cells were prepared by swim-up and capacitated as described by Lishko *et al*. (2011). Whole-cell currents were evoked by 1 s voltage ramps from −80 to +80 mV from a holding potential of 0 mV (before correction for junction potential). As previously described, seals were formed either at the human sperm cytoplasmic droplet located in the neck region in HS solution (Lishko *et al*., 2011). Pipettes were filled with a Cs^+^-based medium (Lishko *et al*., 2011) containing 130 mM Cs-methane sulphonate, 40 mM HEPES, 1 mM Tris–HCl, 3 mM EGTA, 2 mM EDTA, pH adjusted to 7.4 with CsOH. A divalent-free bath solution comprising 140 mM CsMeSO_3_, 40 mM HEPES and 3 mM EGTA (pH 7.4) was used, thus allowing proper recordings of CatSper monovalent currents. 5 µM *p*,*p*′-DDE was added at specific time points. All experiments were performed at 25°C.

### Extended sperm incubations with *p*,*p*′-DDE

After liquefaction, spermatozoa were isolated by density gradient centrifugation (Isolate^®^ Sperm Separation Medium, Irvine Scientific, CA, USA) and allowed to capacitate for at least 3 h at 37°C under 5% CO_2_/95% air. Spermatozoa (10 million/ml) were then exposed to several *p*,*p*′-DDE concentrations (1, 10, 25 and 50 µM) for 3 days at 37°C under 5% CO_2_/95% air in order to mimic the putative continuous exposure to toxicants in the female reproductive tract *in vivo*. Cells were maintained in a phosphate buffered saline (PBS; Invitrogen, Paisley, UK) containing 0.9 mM CaCl_2_, 0.5 mM MgCl_2,_ 5 mM d-glucose, 1.0 mM Na-pyruvate, 10.0 mM Na-lactate, 0.3% (w/v) BSA and 1% (v/v) penicillin/streptomycin, pH 7.2–7.4, according to our formerly described long-standing culture system (Amaral *et al*., 2011). All sperm parameters were assessed daily and medium was changed every day after a 10-min centrifugation at 528*g*. Solvent controls were performed by adding 0.3% (v/v) DMSO.

### Sperm viability

In order to evaluate membrane integrity, spermatozoa were incubated with the LIVE/DEAD Sperm Vitality kit (Molecular Probes) as previously described (Amaral and Ramalho-Santos, 2010). Two hundred spermatozoa were observed in each slide using a Zeiss Axioplan 2 Imaging fluorescence microscope (Carl Zeiss, Göttingen, Germany). Results were displayed as percentage of live spermatozoa relative to the control.

### Acrosomal integrity

Acrosomal integrity was evaluated using the acrosomal content marker *Pisum Sativum* agglutinin coupled to fluorescein isothiocyanate (PSA-FITC), as described elsewhere (Mota *et al*., 2012). The proportion of spermatozoa with intact acrosome was observed under a Zeiss Axioplan 2 Imaging fluorescence microscope and two hundred spermatozoa were scored in each slide. Results are presented as percentage of intact acrosomes relative to the control.

### Statistical analysis

Statistical analysis was carried out using the SPSS version 19.0 software for Windows (SPSS Inc., Chicago, IL, USA). All variables were checked for normal distribution and multiple comparisons were performed by paired *t*-test or one-way analysis of variance (ANOVA). Correlations were performed by the Spearman non-parametric test. Results are expressed as mean% ± SEM. *P* < 0.05 was considered significant.

## Results

### *p*,*p*′-DDE promotes an intracellular Ca^2+^ rise

**Figure 1:**
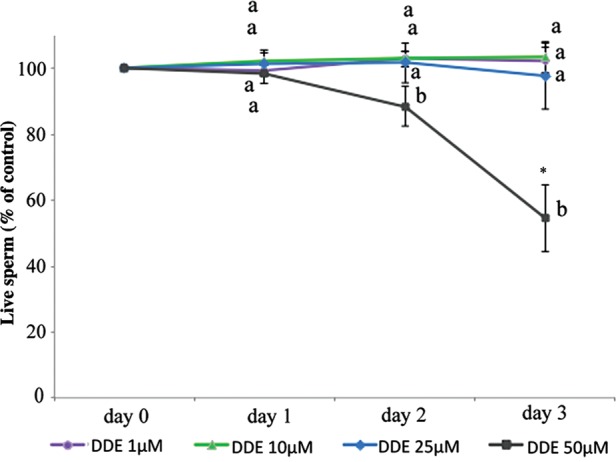
Daily assessment of sperm survival during continuous exposure to *p*,*p*′-DDE concentrations at 37°C and 5% CO_2._ Results represent mean percentage ± SEM relative to control (100 × %live/% live in control), *n* = 5. Asterisk and different letters represent significant differences compared with control and between concentrations, respectively (*P* < 0.05).

**Figure 2:**
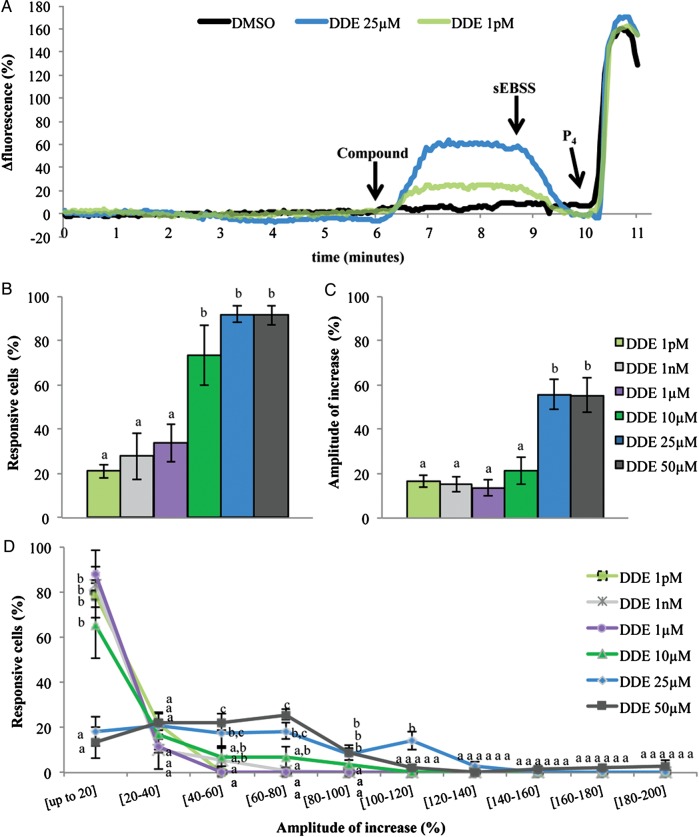
Intracellular Ca^2+^ levels during *p*,*p*′-DDE exposure in human sperm. (**A**) Fluorescence-time traces representing intracellular Ca^2+^ changes in three individual cells exposed to different conditions. DMSO (black trace), 1 pM (green trace) or 25 µM *p*,*p*′-DDE (blue trace) were added after 6 min of perfusion with standard sEBSS. After a further 3 min *p*,*p*′-DDE was washed out by perfusion with fresh sEBSS. Arrows indicate the exact time points in which spermatozoa were bathed with different solutions. P_4_—3.2 µM progesterone. (**B**) Proportion of cells responsive to *p*,*p*′-DDE. (**C**) Magnitude of Ca^2+^ response in responsive cells. (**D**) Amplitude distribution of [Ca^2+^]_i_ increase (significant increase in fluorescence) at each dose tested. Results represent mean percentage ± SEM from 500 cells analyzed individually in a total of five independent experiments for each *p*,*p*′-DDE concentration. Different letters denote statistical differences between concentrations (*P* < 0.05). (EBSS, Earle's balanced salt solution.)

Using a continuous exposure system to better mimic *in vivo* conditions we determined that *p*,*p*′-DDE did not affect sperm viability, except after 3 days of continuous exposure to the highest *p*,*p*′-DDE concentration tested (50 µM; *P* < 0.05, Fig. 1). However, single-cell assessment of [Ca^2+^]_i_ showed that a wide range of *p*,*p*′-DDE concentrations (1 pM–50 µM) caused a clear increase in Ca^2+^ levels within seconds of exposure (when compared with the control), reversible upon sEBSS media washout (Fig. 2A). At 25 and 50 µM of *p*,*p*′-DDE, >91% of spermatozoa showed a significant increase in [Ca^2+^]_i_ (*P* < 0.05, Fig. 2B) and similar mean Ca^2+^ response amplitudes (55.6 ± 6.7 and 55.5 ± 8.1%, respectively; Fig. 2C). *p*,*p*′-DDE was found to be so effective that even at concentrations as low as 1 pM and 1 nM, we observed elevated Ca^2+^ levels in 21.1 ± 3.0 and 28.0 ± 10.65% of cells (*P* < 0.05, Fig. 2B), with mean amplitudes of response of 16.7 ± 2.8 and 15.4 ± 3.6%, respectively (Fig. 2C). Whereas the dose–effect curve for the proportion of responsive cells was roughly sigmoidal, the curve for [Ca^2+^]_i_ response amplitudes appeared biphasic, with markedly greater responses at 25 and 50 µM *p*,*p*′-DDE (Fig. 2B and C). When we analyzed the amplitude distribution of the single-cell responses we observed that from 1 pM to 10 µM *p*,*p*′-DDE most responsive cells showed an increase in fluorescence intensity of up to 20%, but at higher doses the shape of the distribution was completely different, with ‘enhanced’ response amplitudes ranging between 20 and 100% and occasional responses of up to 200% (Fig. 2D). A positive control was included by adding the physiological stimulus progesterone, which causes increased Ca^2+^ levels and triggers AR, to ensure that all samples were responding normally.

### The effect of *p*,*p*′-DDE on [Ca^2+^]_i_ is abolished in low-Ca^2+^ medium

We next evaluated whether *p*,*p*′-DDE effect on human sperm was due to a Ca^2+^ influx from the medium or to the mobilization of intracellular Ca^2+^ stores present in sperm (reviewed in Jimenez-Gonzalez *et al*., 2006; Costello *et al*., 2009). These and subsequent Ca^2+^ imaging experiments were performed with 1 pM and 1 nM *p*,*p*′-DDE, concentrations within the range often found in human reproductive fluids (mean values ranging from 47 pM to 111 nM according to Kumar *et al*., 2000; Dallinga *et al*. 2002; Younglai *et al*., 2002; Pant *et al*., 2004) and also at 25 µM, the minimal saturating concentration for the observed effects on [Ca^2+^]_i_.

**Figure 3:**
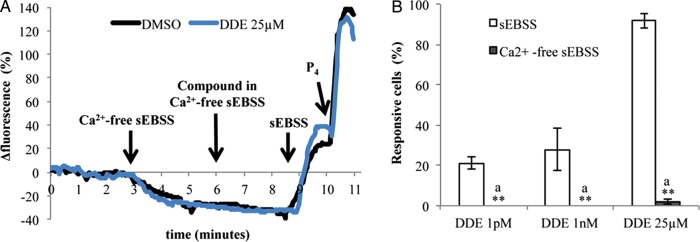
Effect of *p*,*p*′-DDE in a low-Ca^2+^ sEBSS medium (<500 nM). (**A**) Fluorescence-time traces representing intracellular Ca^2+^ changes in two individual cells exposed to different conditions. DMSO (black trace) or 25 µM *p*,*p*′-DDE (blue trace), both diluted in low-Ca^2+^-sEBSS medium, were added after 6 min of perfusion. Arrows represent the exact time points in which spermatozoa were bathed with different solutions. P_4_ —3.2 µM progesterone. (**B**) Percentage of *p*,*p*′-DDE responsive cells. Results represent mean percentage ± SEM from 500 cells evaluated individually in a total of eight independent experiments for each *p*,*p*-DDE dose. Double asterisks correspond to statistical differences between concentrations subjected to different conditions (*P* < 0.01). Similar letters represent lack of statistical significance. (EBSS, Earle's balanced salt solution.)

Perfusion of the recording chamber with low-Ca^2+^ medium (<500 nM) caused an immediate decrease in sperm [Ca^2+^]_i_ that stabilized at a new level within 3 min of exposure and remained unaltered when *p*,*p*′-DDE was added (Fig. 3A). At 1 pM and 1 nM no cells showed [Ca^2+^]_i_ responses (*P*<0.01 compared with experiments in standard sEBSS, Fig. 3B) and at 25 µM *p*,*p*′-DDE only 2.0 ± 1.2% of cells responded with an increase in Ca^2+^ levels (*P* < 0.01 when compared with the 91.9 ± 3.7% of cells in standard sEBSS; Fig. 3B). Furthermore, the magnitude of response provoked by 25 µM *p*,*p*′-DDE was only of 22.8 ± 10.7% compared with the 55.6 ± 6.7% observed in standard sEBSS (*P* < 0.05). When we analyzed the distribution of the single-cell response we found that 72.2 ± 14.7% of cells responded with an increase in fluorescence intensity of up to 20%, resembling the response observed in spermatozoa exposed from 1 pM to 10 µM *p*,*p*′-DDE in standard sEBSS medium. In all these experiments performed in a low-Ca^2+^ medium, when standard sEBSS was returned to the chamber [Ca^2+^]_i_ levels increased as expected, and responded normally to the progesterone stimulus (Fig. 3A).

### *p*,*p*′-DDE effect on [Ca^2+^]_i_ is reversed by CatSper blockers

**Figure 4:**
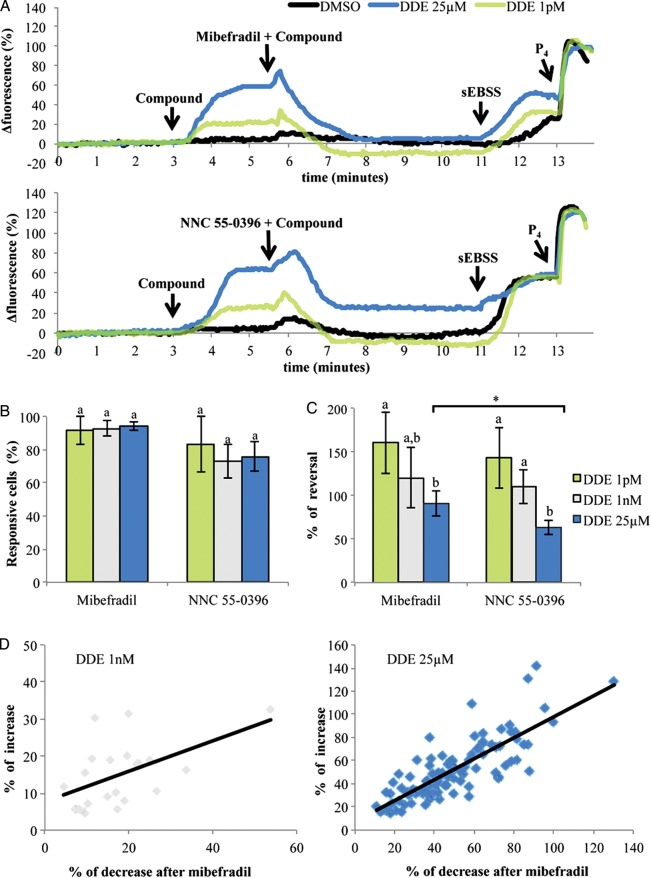
Mibefradil and NNC 55-0396 effects following *p*,*p*′-DDE-induced [Ca^2+^]_i_ rise. (**A**) Fluorescence-time traces representing intracellular Ca^2+^ changes in six individual cells exposed to different conditions. DMSO (black traces), 1 pM (green traces) or 25 µM *p*,*p*′-DDE (blue traces) were added after 3 min of perfusion with standard sEBSS. Thirty micromolars of mibefradil or 10 µM NNC 55-0396 were applied after a further 2.5 min when effects on [Ca^2+^]_i_ had stabilized. Arrows represent the exact time points in which spermatozoa were bathed with different solutions. P_4_ —3.2 µM progesterone. (**B**) Proportion of responsive cells. (**C**) Percentage reversal by mibefradil and NNC 55-0396 of the preceding increase induced by *p*,*p*′-DDE. Mibefradil and NNC 55-0396 alone had no effect (not shown). Results represent mean percentage ± SEM from 500 cells analyzed individually in a total of five independent experiments for each *p*,*p*′-DDE concentration. Different letters denote statistical significance between concentrations within each inhibitor experiments (*P* < 0.05) and asterisk represents statistical differences between the same concentrations exposed to both inhibitors (*P* < 0.05). (**D**) Correlation between amplitudes of the *p*,*p*′-DDE-induced [Ca^2+^]_i_ rise and the subsequent fall in [Ca^2+^]_i_ upon mibefradil application in individual sperm exposed to 1 nM (left panel) or 25 µM *p*,*p*′-DDE (right panel). Significant correlations were found for both 1 nM (*ρ* = 0.492, *P* < 0.05) and 25 µM *p*,*p*′-DDE (*ρ* = 0.804, *P* < 0.001). Each panel shows all cells from a single experiment. (EBSS, Earle's balanced salt solution.)

CatSper, the only Ca^2+^ conductance channel that has been detected in patch-clamped human sperm (Kirichok and Lishko, 2011), is highly promiscuous, activating in response to a wide range of small organic molecules (Brenker *et al*., 2012). In order to investigate whether activation of CatSper might mediate *p*,*p*′-DDE-induced Ca^2+^ influx, we used 30 µM mibefradil and 10 µM NNC 55-0396, both of which inhibit CatSper currents in human sperm (Lishko *et al*., 2011; Strünker *et al*., 2011). Cells were first exposed to *p*,*p*′-DDE (1 pM, 1 nM and 25 µM) to establish Ca^2+^-influx and after a delay of 2.5 min the inhibitors were added in separate experiments (Fig. 4A). Both the drugs caused a transient increase in fluorescence, as previously described (Strünker *et al*., 2011; Brenker *et al*., 2012) which also occurred in control experiments in the absence of *p*,*p*′-DDE (Fig. 4A DMSO trace). However, within few minutes [Ca^2+^]_i_ significantly decreased and stabilized at a new, lower level (Fig. 4A and B). 30 µM mibefradil strongly reversed the effect of *p*,*p*′-DDE in >90% of cells (Fig. 4A–C). This effect was observed at all doses and when mibefradil was applied during 1 pM or 1 nM *p*,*p*′-DDE exposure [Ca^2+^]_i_ decreased below control conditions (*P*> 0.05, Fig. 4A), therefore showing a reversal effect >100% (Fig. 4C). Examination of individual cell responses showed that the magnitudes of the rise in fluorescence caused by *p*,*p*′-DDE and the subsequent decrease upon application of mibefradil were clearly correlated (*P* < 0.05, Fig. 4D), confirming that mibefradil was acting by blocking the effect of *p*,*p*′-DDE.

10 µM NNC 55-0396 also reversed the *p*,*p*′-DDE-induced [Ca^2+^]_i_ rise in most cells (*P* < 0.05; Fig. 4A–C). However, NNC 55-0396 reversal of the [Ca^2+^]_i_ rise caused by 25 µM *p*,*p*′-DDE was only partial when compared with mibefradil (*P* < 0.05; Fig. 4C). Analysis of individual spermatozoa responses showed that, similarly to the effect of mibefradil, the amplitude of the effect of NNC 55-0396 was correlated with the amplitude of the preceding rise induced by *p*,*p*′-DDE (*P* < 0.05, Supplementary data, Fig S1).

### *p*,*p*′-DDE enhances CatSper currents in human sperm

**Figure 5:**
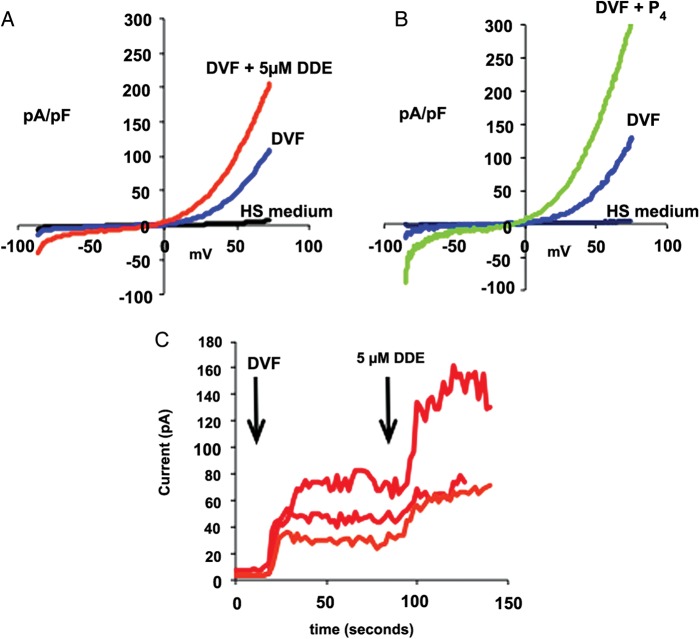
Effect of 5 µM *p*,*p*′-DDE on monovalent CatSper currents in human sperm. (**A**) Example of currents induced by applying a 1 s voltage ramp from −80 to 80 mV to a cell bathed in divalent cation-containing medium (black trace), after superfusion with divalent-free Cs^+^-based medium (DVF; blue trace) and then after application of 5 µM *p*,*p*′-DDE (red trace). (**B**) An example of a similar experiment in which the current was recorded first in divalent cation-containing medium (black trace), then after superfusion with DVF (blue trace) and finally in the presence of 3.2 µM progesterone (P_4_; green trace). (**C**) Time-course of changes in current induced by 5 µM *p*,*p*′-DDE. Current amplitude was quantified using the average current over the last 3 mV of the voltage ramp (77–80 mV). Traces show responses of three different cells. The first arrow shows superfusion with DVF and the second shows application of 5 µM *p*,*p*′-DDE.

The action of *p*,*p*′-DDE on [Ca^2+^]_i_ is mediated by Ca^2+^ influx and can be reversed by CatSper antagonistic drugs, suggesting that this DDT metabolite activates CatSper. To confirm this, we investigated the effect of 5 µM *p*,*p*′-DDE (a concentration that gave detectable [Ca^2+^]_i_ responses in 50.0% of cells but where response amplitude was not ‘enhanced’ as suggested by Fig. 2D) on CatSper in human sperm held under whole-cell clamp. Using divalent-free conditions and Cs methanesulphonate-based bath and pipette media, large CatSper currents, carried by Cs^+^, were induced by 1 s voltage ramps from −80 to +80 mV (Lishko *et al*., 2011). Five micromolars of *p*,*p*′-DDE, increased CatSper current by 116.0 ± 10.0% (*n* = 5; *P* < 0.01) without changing reversal potential or the characteristic outward rectification of the current (Fig. 5A), similarly to the agonistic effect of 3.2 µM progesterone (Fig. 5B). Examination of the time-course of the action of *p*,*p*′-DDE showed that currents increased slowly over a period of 10–20 s and then stabilized (Fig. 5C). In most cells, seals became unstable after 1–2 min and recordings were lost abruptly or after a second rapid rise in current.

### *p*,*p*′-DDE induces spontaneous acrosomal loss

**Figure 6:**
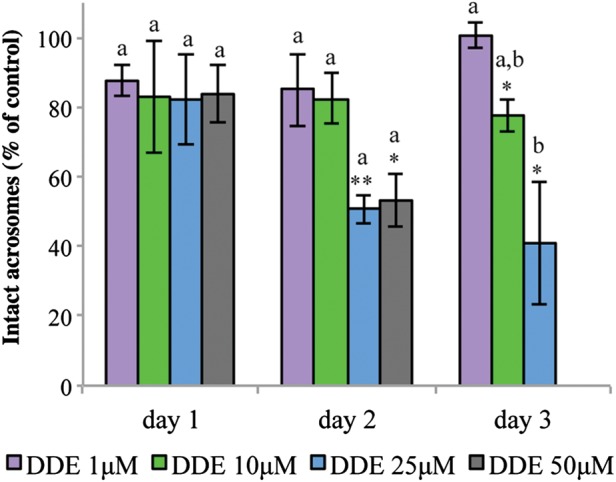
Daily evaluation of acrosomal integrity during continuous exposure to *p*,*p*′-DDE concentrations at 37°C and 5% CO_2_. Results represent mean percentage ± SEM relative to the control (100 × % acrosome intact/% acrosome intact in control), *n* = 6. *P < 0.05 and ** < 0.01 denote differences towards DMSO and different letters between concentrations (*P* < 0.05).

To evaluate if changes in [Ca^2+^]_i_ could affect sperm function, acrosomal integrity was assessed (Fig. 6). Although *p*,*p*′-DDE did not affect the percentage of intact acrosomes at day 1 (*P* > 0.05), 25 and 50 µM *p*,*p*′-DDE significantly reduced acrosomal integrity after 2 days of exposure (*P* < 0.01 and 0.05, respectively). This effect was further observed at Day 3 for both 25 and 10 µM *p*,*p*′-DDE (*P* < 0.05). No differences were observed at 1 µM *p*,*p*′-DDE in this 3-day long approach. Differences between concentrations were only found at day 3 for 1 and 25 µM *p*,*p*′-DDE (*P* < 0.05, Fig. 6). Due to the strong decrease observed in sperm viability (Fig. 1), acrosomal integrity was not evaluated following 3 days of continuous exposure to 50 µM *p*,*p*′-DDE (Fig. 6), given that this data could be misleading, reflecting the loss of viability. It should also be noted that, while subtle changes in Ca^2+^ levels were detected in previous experiments, acrosomal integrity monitored here reflects an all-or-nothing measurement, and relevant changes in the sperm secretory vesicle may occur much earlier. Further experiments, also using longer incubation periods with CatSper inhibitors, are warranted to further clarify this issue.

## Discussion

Several studies have focused on the likely genomic effects of *p*,*p*′-DDE on male fertility (You *et al*., 1998; Loeffler and Peterson, 1999; Ayotte *et al*., 2001; Hauser *et al*., 2002, 2003; Rignell-Hydbom *et al*., 2005a, 2005b; De Jager *et al*., 2006; Toft *et al*., 2006; Stronati *et al*., 2006; Aneck-Hahn *et al*., 2007), without exploring possible rapid non-genomic actions on human sperm. This is especially important as sperm can be exposed to *p*,*p*′-DDE through seminal fluid and/or in secretions in the female reproductive tract (Kumar *et al*., 2000; Younglai *et al*., 2002; Pant *et al*., 2004), where male gametes can survive for several days (Amaral *et al*., 2011). Additionally, spermatozoa are excellent models for the analysis of non-genomic effects of environmental pollutants/endocrine disruptors since they are transcriptionally inactive, and thus genomic effects will not confound the analysis. We found that *p*,*p*′-DDE consistently promoted a [Ca^2+^]_i_ rise in human sperm, as observed by single-cell imaging. High concentrations of *p*,*p*′-DDE caused a large and rapid rise in [Ca^2+^]_i_ fluorescence which increased by up to 200%, but even concentrations as low as 1 pM and 1 nM induced significant responses.

Effects on cytosolic Ca^2+^ levels after exposure to several toxicants, including *p*,*p*′-DDE, have been reported in many cell types, apparently mimicking the action of steroids (Ruehlmann *et al*., 1998; Nadal *et al*., 2000; Younglai *et al*., 2004, 2006; Wu *et al*., 2006), but dose dependence and magnitude of the effect vary greatly. *p*,*p*′-DDE and other pesticides such as kepone, methoxychlor and the isomer *o*,*p*-DDE were found to increase cytosolic Ca^2+^ levels in granulosa-lutein and HUVE cells (Younglai *et al*., 2004; Wu *et al*., 2006), although in granulosa-lutein cells the Ca^2+^ changes induced by methoxychlor and *o*,*p*-DDE were not as clear or consistent as those induced by kepone (Wu *et al*., 2006). Furthermore, methoxychlor at high concentrations (2.8–280 µM) failed to induce changes in Ca^2+^ levels (Wu *et al*., 2006). In fact, contrary to the sigmoid curve of dose–response found in this study, the effect of methoxychlor is another example of a non-classical response, showing an inverse U-shaped curve (Wu *et al*., 2006). The traditional dose–response effect observed in many toxicological studies is not always applicable, especially when environmental toxicants acting as endocrine disruptors are involved (Krimsky, 2001). In mouse β pancreatic cells bisphenol A (BPA), diethylstilbestrol and *o*,*p*′-DDT increased the frequency of glucose-provoked [Ca^2+^]_i_ fluctuations (Ruehlmann *et al*., 1998). A similar response was observed at pico- and nanomolar concentrations in a GH3/B6 pituitary cell line exposed to *o*,*p*-DDE (Wozniak *et al*., 2005), showing the concerning extensive range of action of these endocrine disruptors in the environment. Accordingly, although 10 µM *p*,*p*′-DDE failed to affect [Ca^2+^]_i_ in rat myometrial smooth muscle cells, 50 and 100 µM *p*,*p*′-DDE-induced [Ca^2+^]_i_ rise by 586% and 921%, respectively (Juberg *et al*., 1995), effects far greater than those reported here.

To further assess *p*,*p*′-DDE mechanisms of action in human sperm we exposed cells to the compound in a low-Ca^2+^ medium. Under these conditions the effect was largely abolished, showing that *p*,*p*′-DDE mainly promotes Ca^2+^ influx at the plasma membrane. Intriguingly, although higher concentrations of *p*,*p*′-DDE resulted in larger [Ca^2+^]_i_ signals (Fig. 2C), this effect apparently occurred by ‘recruitment’ of a larger ‘type’ of Ca^2+^ signal (Fig. 2D). This may possibly reflect a secondary release of stored Ca^2+^ downstream of Ca^2+^ influx (Harper *et al*., 2004). To further explore which plasma membrane Ca^2+^ channel(s) were involved, a pharmacological approach was used. In mouse and human sperm, CatSper is believed to be the principal plasma membrane Ca^2+^ channel (Kirichok *et al*., 2006; Qi *et al*., 2007; Smith *et al*., 2013). Using the Catsper blockers mibefradil and NNC 55-0396 (Lishko *et al*., 2011; Strünker *et al*., 2011), we observed a strong suppression of the *p*,*p*′-DDE-induced Ca^2+^ increase in the large majority of cells. NNC 55-0396, the putatively more potent Catsper inhibitor (Lishko *et al*., 2011) induced a lower decrease of Ca^2+^ levels at 25 µM *p*,*p*′-DDE when compared with mibefradil, but this may reflect the significant rise in [Ca^2+^]_i_ caused by NNC 55-0396 itself (Strünker *et al*., 2011). We further confirmed *p*,*p*′-DDE action on CatSper using whole-cell patch-clamp recordings with divalent cation-free bath and pipette solutions where Cs^+^ was the only permeably cation, conditions under which the large monovalent currents show CatSper activity (Kirichok *et al*., 2006; Lishko *et al*., 2011; Strünker *et al*., 2011). Treatment with *p*,*p*′-DDE caused instability and ultimately loss of the seal within 1–2 min, an effect that is apparently related to patch formation and/or the recording conditions used, since cell viability was not affected (Fig. 1). It has been shown by patch clamp that human sperm CatSper currents are powerfully potentiated by progesterone (Kirichok and Lishko, 2011; Strünker *et al*., 2011), whereas the steroid had no effect on currents in sperm from an infertile CatSper-deficient patient (Smith *et al*., 2013), suggesting that CatSper is central to the non-genomic action of the steroid. The high potency of *p*,*p*′-DDE in elevating [Ca^2+^]_i_ in human sperm may therefore reflect a steroid-like effect and *p*,*p*′-DDE might even bind the same activating site as progesterone and thus promote Ca^2+^ influx, although the sustained nature of the *p*,*p*′-DDE-induced signal does not resemble the biphasic [Ca^2+^]_i_ elevation induced by progesterone. Alternatively, this action of *p*,*p*′-DDE may reflect a more general feature of CatSper. In addition to progesterone the channel is activated by membrane potential, pH_i_, prostaglandins, odorants and other small organic molecules (Lishko *et al*., 2011; Strünker *et al*., 2011; Brenker *et al*., 2012), apparently acting as a polymodal sensor upon which diverse stimuli converge to generate [Ca^2+^]_i_ signals in sperm. The promiscuous nature of the channel, though apparently important for detection of cues in the female tract (Brenker *et al*., 2012), may render sperm sensitive to organochlorine pollutants such as *p*,*p*′-DDE.

After observing these intracellular Ca^2+^ changes, we suspected that AR, a strongly Ca^2+^-dependent event, would be affected. In fact, by mimicking the female reproductive tract conditions, where sperm can be maintained for days, potentially with constant *p*,*p*′-DDE exposure, we found decreased acrosomal integrity suggesting the induction of spontaneous AR following 2 and 3 days of exposure. Although other pathways may certainly be involved, and further studies are warranted, we hypothesize that this effect was possibly achieved by sustained Ca^2+^ overload promoted by *p*,*p*′-DDE. Elevated *p*,*p*′-DDE concentrations not only promoted [Ca^2+^]_i_ rise in a higher percentage of cells with higher magnitudes of response but also induced acrosomal loss earlier in time. In contrast, since 10 µM *p*,*p*′-DDE induced smaller magnitudes of response a decrease in acrosomal integrity was only detected after 3 days of exposure (Figs 2 and 6). In accordance, an environmentally relevant mixture containing *p*,*p*′-DDE was found to induce increased [Ca^2+^]_i_ and potentiated spontaneous AR rates in boar sperm (Campagna *et al*., 2009). Although the authors did not explore which was the source responsible for the observed higher Ca^2+^ levels, they suggested that this mixture could modify the sperm plasma membrane, allowing non-regulated Ca^2+^ entry that would finally lead to AR, thus lowering sperm survival, among other effects (Campagna *et al*., 2009). In contrast, the organochlorine pesticide lindane was found to inhibit spontaneous AR in human sperm (Silvestroni and Pallesch, 1999). This compound was able to quickly and transiently depolarize the sperm plasma membrane, opening channels and causing an increase in intracellular Ca^2+^ levels, but probably by inducing biophysical changes on the sperm surface (Silvestroni *et al*., 1997) and AR was reduced (Silvestroni and Pallesch, 1999). On the contrary, both BPA and octylphenol failed at inducing AR and modifying [Ca^2+^]_i_ in human sperm (Luconi *et al*., 2001). Recently, we observed the same complete lack of effects on AR and [Ca^2+^]_i_, among other functional parameters, in human sperm directly exposed to the classical dioxin 2,3,7,8-tetrachorodibenzo-*p*-dioxin (TCDD; Mota *et al*., 2012), using the same approach and compound solvent, serving, in essence, as a negative control for the data presented here. In general, these data clearly support the involvement of different mechanisms of action through which endocrine disruptors exert their effects, but the highly promiscuous nature of CatSper may cause sperm sensitivity to several compounds that interact with key site(s) on the channel.

In this study, prolonged *p*,*p*′-DDE exposure was shown to decrease sperm survival, although only at the highest concentration tested, at day 3. Overall, these findings suggest that the *p*,*p*′-DDE-induced [Ca^2+^]_i_ rise may prematurely trigger acrosomal loss (either via spontaneous AR or damage to sperm membranes) and affect sperm viability long before they reach the oocyte, thus adversely affecting male fertility. *p*,*p*′-DDE concentrations in follicular fluid have already been correlated with failed fertilization (Younglai *et al*., 2002) and described as being higher in semen from infertile patients (Pant *et al*., 2004), suggesting an important role of *p*,*p*′-DDE in human (in)fertility.

## Conclusion

Even at concentrations found in reproductive fluids, *p*,*p*′-DDE was able to induce a rise in [Ca^2+^]_i_ in human sperm through a novel non-genomic mechanism involving the opening of the sperm-specific cation channel CatSper and consequently affected acrosome status and sperm survival, ultimately compromising male fertility.

## Supplementary data

Supplementary data are available at http://humrep.oxfordjournals.org/.

## Authors’ roles

J.R.S., R.S.T., S.J.P., S.M.W. and C.L.B. established the concept and design. R.S.T. and S.M. acquired data. R.S.T., S.J.P. and J.R.S. wrote the paper and all authors contributed to the analysis and interpretation of the results, drafting, revising and approving the article.

## Funding

This work was supported by the Portuguese National Science Foundation (FCT) through a grant attributed to the CNC Institution (PEst-C/SAU/LA0001/2011) and a Wellcome Trust Grant #86470 provided to S.J.P. and C.L.R.B. S.M. was supported by the Infertility Research Trust. R.S.T. is the recipient of a PhD fellowship from FCT (SFRH/BD/46002/2008). Funding to pay the Open Access publication charges for this article was provided by The Wellcome Trust.

## Conflict of interest

None declared.

## Supplementary Material

Supplementary Data
