# Supplementary material for: p,p′-DDE activates CatSper and compromises human sperm function at environmentally relevant concentrations
Source: Hum Reprod. 2013 Sep 24;28(12):3167–77. doi: 10.1093/humrep/det372 (PMC3829580; doi:10.1093/humrep/det372)
Supplement: Supplementary Data [file supp_28_12_3167__index.html]

p,p′-DDE activates CatSper and compromises human sperm function at environmentally relevant concentrations — Supplementary Data 

# *p*,*p*′-DDE activates CatSper and compromises human sperm function at environmentally relevant concentrations

## Supplementary Data

Supplementary Data

**Files in this Data Supplement:**

- Supplementary Data - Pdf file
