# Supplementary material for: p,p′-DDE activates CatSper and compromises human sperm function at environmentally relevant concentrations
Source: Hum Reprod. 2013 Sep 24;28(12):3167–77. doi: 10.1093/humrep/det372 (PMC3829580; doi:10.1093/humrep/det372)
Supplement: Supplementary Data [file supp_det372_det372supp.pdf]

Human Reproduction, Vol.0, No.0 pp. 1–1, 2013
Advanced Access publication on XX, XXXX doi:10.1093/humrep/det372

human reproduction

SUPPLEMENTARY DATA

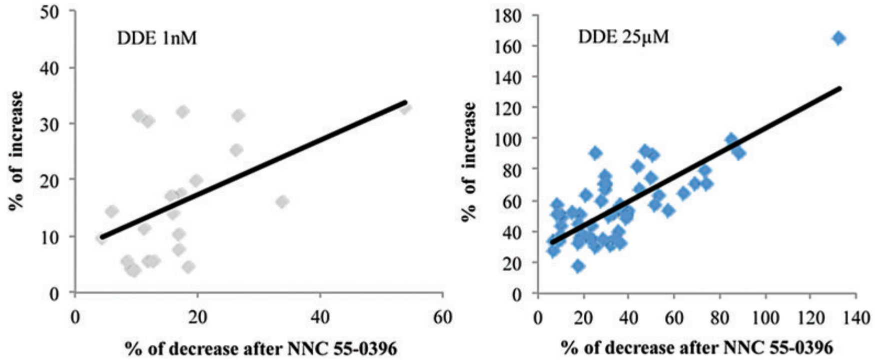

Supplementary Figure 1 Correlation between amplitudes of the *p,p*′-DDE-induced $[Ca^{2+}]_i$ rise and subsequent fall in $[Ca^{2+}]_i$ upon NNC 55-0396 application in individual sperm cells exposed to 1 nM (left panel) or 25 μM *p,p*′-DDE (right panel). Significant correlations were found for both 1 nM ($\rho = 0.506$, $P < 0.05$) and 25 μM *p,p*′-DDE ($\rho = 0.635$, $P < 0.001$). Each panel shows all cells from a single experiment.

© The Author 2013. Published by Oxford University Press on behalf of the European Society of Human Reproduction and Embryology. All rights reserved. For Permissions, please email: journals.permissions@oup.com
